# Convert your favorite protein modeling program into a mutation predictor: “MODICT”

**DOI:** 10.1186/s12859-016-1286-0

**Published:** 2016-10-19

**Authors:** Ibrahim Tanyalcin, Katrien Stouffs, Dorien Daneels, Carla Al Assaf, Willy Lissens, Anna Jansen, Alexander Gheldof

**Affiliations:** 1 Center for Medical Genetics, UZ Brussel, Laarbeeklaan 101, Brussel, 1090 Belgium; 2 Neurogenetics Research Group, Reproduction Genetics and Regenerative Medicine Research Group, Vrije Universiteit Brussel (VUB), Laarbeeklaan 101, Brussel, 1090 Belgium; 3 Center for Medical Genetics, Reproduction and Genetics, Reproduction Genetics and Regenerative Medicine, Vrije Universiteit Brussel (VUB), UZ Brussel, Laarbeeklaan 101, Brussel, 1090 Belgium; 4 Pediatric Neurology Unit, Department of Pediatrics, UZ Brussel, Laarbeeklaan 101, Brussel, 1090 Belgium; 5 Center for Human Genetics, KU Leuven and University Hospitals Leuven, Herestraat 49, Leuven, 3000 Belgium

**Keywords:** Prediction, 3D protein model, Bioinformatics

## Abstract

**Background:**

Predict whether a mutation is deleterious based on the custom 3D model of a protein.

**Results:**

We have developed modict, a mutation prediction tool which is based on per residue rmsd (root mean square deviation) values of superimposed 3D protein models. Our mathematical algorithm was tested for 42 described mutations in multiple genes including renin (*REN*), beta-tubulin (*TUBB2B*), biotinidase (*BTD*), sphingomyelin phosphodiesterase-1 (*SMPD1*), phenylalanine hydroxylase (*PAH*) and medium chain Acyl-Coa dehydrogenase (*ACADM*). Moreover, modict scores corresponded to experimentally verified residual enzyme activities in mutated biotinidase, phenylalanine hydroxylase and medium chain Acyl-CoA dehydrogenase. Several commercially available prediction algorithms were tested and results were compared. The modict perl package and the manual can be downloaded from https://github.com/IbrahimTanyalcin/MODICT.

**Conclusions:**

We show here that modict is capable tool for mutation effect prediction at the protein level, using superimposed 3D protein models instead of sequence based algorithms used by polyphen and sift.

**Electronic supplementary material:**

The online version of this article (doi:10.1186/s12859-016-1286-0) contains supplementary material, which is available to authorized users.

## Background

### State of the art

As next generation sequencing (NGS) is advancing the field of molecular biology today, more human protein variants are identified than ever before. One of the greatest challenges in this field is to be able to predict whether the detected variants are real disease-causing changes underlying the patients condition.

The current concept of mutation effect prediction heavily depends on the composite algorithms that mainly implement a sequence-based BLAST search that tries to identify a number of similar protein sequences above a preset threshold, then relate and combine several other parameters such as PSIC (Position-Specific Independent Counts), known three-dimensional (3D) structures of similar proteins, surface area, *β*-factor and atomic contacts. Some available algorithms (e.g.POLYPHEN 2, [1]) use all above whereas others use either a portion or a more diverse set of parameters (e.g.SIFT ([2]), MUTATION TASTER ([3]), PROVEAN ([4]). There are also other algorithms regarding prediction of protein stability such as I-MUTANT and POP-MUSIC which gives results in means of *ΔΔG* [5, 6]. I-MUTANT can both work with sequences and structures from protein data bank (http://www.rcsb.org/, [7]) with temperature and pH optional parameters. POP-MUSIC works with statistical potentials extracted from a test database of known protein structures. Another program, PHD-SNP is sequence based prediction tool that utilizes support vector machines [8]. Since these algorithms take into account non-mutually exclusive (non-orthogonal) features, the method to correctly combine the results to derive a conclusive output remains a challenge. One recently described method uses weighted means obtained from false positive rates and false negative rates of each distinct algorithm to approach a consensus score (Condel, [9]). Even after utilizing cancer-trained methods, such integration of scores were not able to correctly classify all variants [10, 11].

### Hypothesis and problem definition

A high percentage of genomic variants in protein-coding genes were shown to modify the tertiary structure of the coded protein sequence. These structural modifications can be predicted by comparing the 3D structures of the wild type and mutant protein (.pdb files). The 3D structures are generated in commercial or academic-only servers and software (I-TASSER, [12, 13], SWISS-MODEL, [14], MODELLER, [15], YASARA http://www.yasara.org/, [16]) by supplying the raw amino acid sequences in fasta format.

Stability of the proteins depend on many variables such as solvent, temperature and foremost the amino acid sequence. Even a small change in the molecular content, for instance a single nucleotide variation can entirely change the tertiary structure or protein stability [17, 18]. For instance, Wang and Moult have shown that 83 % of a large set of disease causing mutations result in disruption of protein stability [19]. In general, hydrophobic interactions and a network of hydrogen bonds stabilize the folded state of protein [20]. Point mutations (SNVs) can disrupt this folded state by altering hydrophobic interactions, introduction of charged residues into buried sites or breaking beta sheets [19, 21, 22]. As a consequence of the structural changes, protein-protein interactions are also affected by the point mutations [23].

We propose a methodology wherein we perform in silico protein modeling of both the wildtype and mutated protein and where we subsequently calculate the difference in overall 3D structure between the two. This is done by measuring the physical distance between the corresponding residues of the two models after superimposition. Our proposal is that the larger the average distance, the higher the propensity the mutation has to disturb the protein functionality and thus to be pathogenic.

We have derived a simple algorithm called MODICT to predict the effect of mutations on the structure of the protein. It is complementary to the protein modeling tools mentioned above, as it requires the 3D protein structures predicted by these tools. The algorithm takes into account the global structural changes in the 3D protein model. These structural changes are measured in means of the change in **R**oot **M**ean **S**quare **D**eviation (*Δ* RMSD) per residue.

## Methods

### Algorithm

Let *A*_*i*_ denote the RMSD value of a given amino acid at *i* ^*th*^ position resulting from comparison of two models in a cartesian space defined by *V*(*i*,*A*_*i*_) (in other words, *A*_*i*_ is the distance between the same residue *i* of 2 superimposed models which are wildtype and mutated). The distance between two amino acids is based on C-alpha by default which can be easily modified if needed. Assuming the entire length of a protein with N residues is 1 (arbitrarily set), then the **i**ntegral of **s**tep **f**unction (*ISF*) between two consecutive amino acids can be approximated by:

1 $$  \text{ISF} \overset{\mathsf{def}}{=} \frac{A_{i} + A_{i+1}}{2} \cdot \frac{1}{N} \cdot 2 = \frac{A_{i} + A_{i+1}}{N}\quad i \in \left(1,3,5\dots \right)  $$

The aim of *ISF* is to obtain a surrogate measure of how much a given region in a protein molecule has moved away from the original conformation with respect to wildtype protein. If a given domain is enclosed by an interval of *i* ^*th*^ and *j* ^*th*^ amino acid residues then the *ISF* spanning this domain can be expressed as:

2 $$  \text{ISF}_{i,j} \overset{\mathsf{def}}{=} \sum\limits_{n = i}^{j} \left(\frac{A_{i} + A_{i+1}}{N} \cdot W_{i} \cdot C_{i} \right)\quad i \in \left(1,3,5\dots j \right)  $$

where *W*_*i*_ and *C*_*i*_ denote optional weight and conservation scores respectively which are usually provided by the training and iteration modules (users can attain as well). These values are optional and a default fallback value is given if they are not provided (See “MODICT methodology” section for more information, “Btd p.H447R and p.R209C and Tubb2b p.A248V and p.R380L” sections for an example and Section 5.2 in the MODICT manual for the effect of scaling of scores). Of course the aforementioned *ISF* does not solely result from the mutation. A background value can be expressed in terms of overall RMSD ($\overline {\textsc {rmsd}}$;generated by SWISS-MODEL):

3 $$  \mathrm{B}_{i,j} \overset{\mathsf{def}}{=} \frac{\overline{\textsc{rmsd}}}{N} \cdot \left(j-i+1\right) \cdot W_{i} \cdot C_{i}\quad i \in \left(1,3,5\dots j \right)  $$

The aforementioned background value allows one to construct a threshold where above this threshold a given RMSD value can be considered significant. A total (in unit area) can be defined from Eqs. 2 and 3:

4 $$  \sum \textsc{total} \overset{\mathsf{def}}{=} \sum \textsc{isf} + \sum \textsc{b}  $$

Above formula is a generalization for multiple domains. In case there is only one domain between residues *i* and *j*, than the total area simply is ISF_*i*,*j*_+B_*i*,*j*_. A raw score (*Γ*) can be expressed in terms of:

5 $$  \Gamma \overset{\mathsf{def}}{=} \sum \textsc{total} \cdot \frac{\frac{\sum \textsc{isf}}{\sum \textsc{total}}}{\sqrt{\left({\frac{\sum \textsc{isf}}{\sum \textsc{total}}}\right)^{2}+\left({\frac{\sum \textsc{b}}{\textsc{total}}}\right)^{2}}} \cdot \frac{1}{2}  $$

It is noteworthy that for a given interval, *ISF* and *B* are not guaranteed to be equal, even if the regions taken into consideration spans the entire protein. While *ISF* is obtained from per residue RMSD, *B* is obtained from $\overline {\textsc {rmsd}}$. *ISF*/*TOTAL* and *B*/*TOTAL* should be considered as 2 orthogonal vectors. MODICT is designed to work with specific protein domains where i and j designate the start and end of a domain. For MODICT to perform optimal, it is important that the domains which are most critical for the functionality of the protein are chosen. This can be literature findings or can be predicted by the iteration script which is included in the software package (see “Training and iteration” section).

The difference (*δ*) between Eqs. 2 and 3 is important to discern background signal from actual effect:

6 $$  \delta_{i,j} = \textsc{isf}_{i,j} - \textsc{b}_{i,j}  $$

The significance (*γ*) of the difference depends on the length of the domain and the standard deviation of the individual RMSD values:

7 $$  \gamma_{i,j} \overset{\mathsf{def}}{=} Z_{\left(1-\frac{(j-i+1)}{N}\right)} \cdot \frac{\sigma_{\textsc{rmsd}}}{N} \cdot (j-i+1)  $$

where *Z*_*x*_ denotes the Z score of (100·*x*)^*th*^ percentile and *σ* denotes the standard deviation. Assuming that the RMSD values are distributed in a Gaussian distribution, the Z-score derived significance score gives an idea about how much of the domain residues account for the large RMSD values. From Eqs. 6 and 7, a coefficient of significance (*κ*) can be defined:

8 $$  \kappa \overset{\mathsf{def}}{=} \frac{\left(1+\frac{\sum \delta - \sum \gamma}{|\sum \delta|+|\sum \gamma|}\right)}{2}  $$

In the Eq. 8 above, $\sum \delta $ or $\sum \gamma $ denotes the total sum of *δ* or *γ* between all specified domain intervals such as *δ*_*i*,*j*_+*δ*_*m*,*n*_+*δ*_*u*,*w*_…. Equations 5 and 8 can be combined to express a final score:

9 $$  Final Score \overset{\mathsf{def}}{=} \Gamma \cdot \kappa  $$

The criteria of evaluating the score can be performed via 2 different approaches as outlined in “MODICT methodology” section and Additional file 1: Section S1.2. In a fraction of cases, comparison of MODICT scores requires calculating thresholds and these thresholds are calculated via a *K* parameter. Beware that this is not the same coefficient as in Eq. 8. This parameter is a measure of the highest *p*-value attainable with a given accuracy. The *K* parameter is calculated from known list of mutations listed in Additional file 1: Table S1. For more information for the usage of this parameter refer to Additional file 1: Section S1.2.

### MODICT methodology

The algorithm of MODICT is based on rmsd values of superimposed wildtype and mutant proteins. For calculating, RMSD values, a 3D protein model is required of both the wildtype and mutant case, which is calculated by using the I-TASSER and PHYRE2 servers. After construction of the 3D models, the generated pdb files are used as input for a script included in MODICT which will extract the necessary RMSD values. The mutated models should not result from a mere substitution of residues on a pdb viewer, they should be generated using modeling servers or self implemented pipeline of molecular dynamics instead. The user is given the freedom to choose between different modeling servers or self implemented pipeline. Some of the commercially available servers provide only homology modeling, whereas some others will combine homology modeling and ab initio modeling.

For the purpose of testing MODICT, amino acid sequence of wildtype and mutant renin, Tubb2b, Btd and Smpd1 proteins (UNIPROT ID: P00797, Q9BVA1, P43251, P17405) were submitted to the automated I-TASSER and PHYRE2 servers. PAH and ACADM (Tables 1 and 2) were submitted to the automated PHYRE2 server. For further details on specific settings, see Additional file 1: Section S1.1. MODICT can be supplied with optional weight (min:0,default:10) and conservation(min:0,max:11,default:1) scores which are both array vectors (single number per line in a text file). Multiplying all entries of the weight and conservation file by a constant does not change the result. Both files are optional and not mandatory for MODICT to work. However, they can be used to give higher priority to certain regions. The default set up attains 1 to conservation and 10 to weight scores.

**Table 1:** Mutations in *PAH*

Mutation	Residual	Score
	activity (%)	(Higher means more deleterious)
Y414C	28	0.112
R241C	25	0.136
A403V	32	0.125
R261Q	30	0.071
E390G	75	0.086
R68S	98	0.157
I65T	29	0.153
V245A	50	0.126
L48S	39	0.247
F39L	96	0.136
D415N	72	0.072
A395P	15	0.139
A104D	26	0.091
R408Q	55	0.063
P211T	72	0.185
V388M	43	0.15
R241H	23	0.131
I306V	39	0.161

Mutations in PAH with their residual enzyme activity and modict scores are listed. The values listed are expressed in percentages of residual enzyme activity with respect to wildtype enzyme activity. modict scores are generated taking into account the catalytic domain (143–410; [54]). As outlined in “Results” section, when more than or equal to 3 mutations with enzymatic activities are present, the first method can be used where the correlation between the textscmodict scores and the enzymatic activities are measured. Since higher modict scores are more deleterious, one would expect a negative correlation between modict scores and enzyme activity. The interpretation of these results are explained in more detail in Fig. 6

**Table 2:** Mutations in *ACADM*

Mutation pair	Residual activity (%)	Score
K329E/I78T	0	46.5
K329E/M328V	0	59.5
K329E/D345Y	3	57
K329E/M155T	3	51
K329E/K329E	5	53
K329E/L409F	6	56.5
Y337S/Y337S	8	56
G267R/G267R	15	55
K329E/R206C	12.5	59.5
M326T/I233T	15	57
G267R/K178T	20	48
G267R/Y67H	30	47.5
K329E/Y67H	35	46.5
K329E/E43K	60	53.5

Mutation pairs in ACADM with their residual enzyme activity and modict scores are listed. The values listed are expressed in percentages of residual enzyme activity with respect to wildtype enzyme activity. The residual enzyme activities are adapted from Sturm et. al., Fig. 11. modict scores are generated taking into account the main chain (26–421; Uniprot ID, ACADM_HUMAN; [51, 52]). Similar to Table 1, interpretation of these results are detailed in Fig. 5

Conservation scores are generated by aligning reviewed sequences of the protein of interest in different species from UniProt ([24]). It is a simple text file of one conservation score per line and generated using the JALVIEW utility.

MODICT requires a user generated per-residue rmsd file as well. We have developed a script which can be supplied to swiss-pdb. This script extracts the rmsd values from superimposed WT (wildtype) and MT (mutated).pdb files to a file.

**Fig. 1:**
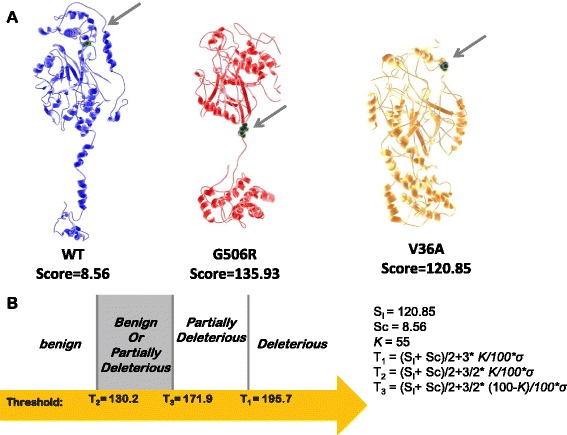
Classification of **S** **m** **p** **d**1^*G*506*R*^. **a**. Wildtype (*blue*), *Smpd*1^*G*506*R*^ (*red*) and *Smpd*1^*V*36*A*^ (*orange*) models are shown. The original position of glycine in wildtype, the substitution site in *Smpd*1^*G*506*R*^ and the alanine 36 in *Smpd*1^*V*36*A*^ are marked with gray arrows. Models have been further refined using the MODREFINER. A negative control score was generated by superimposing the refined wildtype on the initial wildtype whereas a known benign score was generated by superimposing the refined *Smpd*1^*V*36*A*^ on the initial wildtype. A score for the test mutation was generated in the same manner. MODICT scores were generated taking into account the entire backbone (residues 1-629). **b**. Thresholds were calculated as shown in the right and the G506R mutation was classified based on the calculated score bracket as shown in the left. The value of kappa can be updated using the roc.pl script. (*σ*=*standard* *deviation* *of* *S*_*I*_ *and* *S*_*C*_)

MODICT score interpretation makes use of a negative and positive control. As negative control, a superimposition between the wildtype protein and a refined model of the same wildtype protein is used (in some cases, a known benign mutation can also be used instead of refined wildtype, see “ROC curve generation” section and Additional file 1: Section S1.2). For the positive control, superimposition between the wildtype protein and a known pathogenic variant can be used. The scores for the negative and positive control can as such be used as a scale for the MODICT result of the protein variant of interest. A more mathematical approach to MODICT score interpretation is given in “Tubb2b p.A248V and p.R380L” section, Additional file 1: Sections S1.2 and S1.3 and Fig. 1.

### Training and iteration

**Fig. 2:**
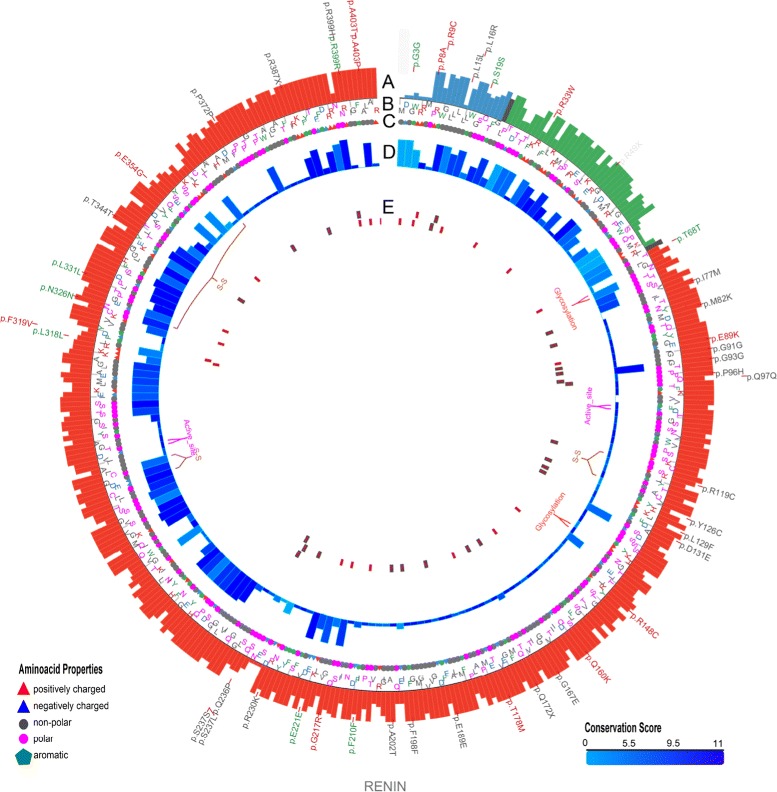
Plot showing conformational differences in *renin* ^*C*20*R*^. Outermost layer indicates reported SNVs (Single Nucleotide Variants; gray, not validated; red, non-synonymous; green, synonymous) from dbSNP 138. **a**. Conservation scores represented as a histogram (blue, signal peptide; green, propeptide; red, domain). These values are generated as described in section 5 and are not related to MODICT score. **b** and **c**. Amino acid sequences with residues colored according to their property (positively charged, *red triangle*; negatively charged, *blue triangle*, non-polar, gray circle; polar, pink circle; aromatic ring, green hexagon). **d**. Iterative MODICT scores of individual residue pairs (algorithm, Eq.1) resulting from comparison with *renin* ^*WT*^ and *renin* ^*R*33*W*^. Each *blue* histogram bin designates the contribution of a residue pair to the overall MODICT score (Higher bars mean more contribution as well as more the adverse effect of that residue pair on structural stability). These histogram bins are generated by iterative MODICT algorithm and are colored according to conservation. **e**. Important regions, SNVs and Indels (insertion-deletions) are marked with boxes. *Red* boxes represent SNVs whereas *pink* boxes represent Indels. *Gray* bordered boxes represent unvalidated changes. (S-S = disulphide bond)

**Fig. 3:**
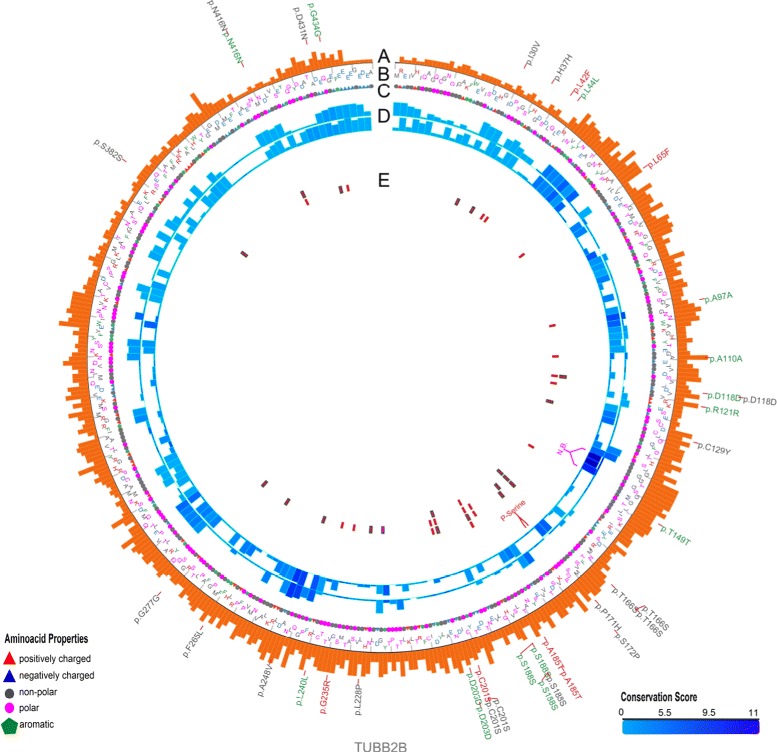
Plot showing conformational differences in *Tubb*2*b* ^*A*248*V*^ and *Tubb*2*b* ^*R*380*L*^. Outermost layer indicates reported SNVs (gray, not validated; red, non-synonymous; green, synonymous) from dbSNP. **a**. Conservation scores represented as a histogram. These values are generated as described in “MODICT methodology” section and are not related to MODICT score. **b** and **c**. Amino acid sequences with residues colored according to their property (positively charged, *red triangle*; negatively charged, *blue triangle*, non-polar, gray circle; polar, pink circle; aromatic ring, green hexagon). **d**. Iterative MODICT scores of individual residue pairs (algorithm, Eq. 1) resulting from comparison with *Tubb*2*b* ^*WT*^. Top layer belongs to *Tubb*2*b* ^*A*248*V*^ whereas bottom layer belongs to *Tubb*2*b* ^*R*380*L*^. Each *blue* histogram bin designates the contribution of a residue pair to the overall MODICT score (Higher bars mean more contribution as well as more the adverse effect of that residue pair on structural stability). These histogram bins are generated by iterative MODICT algorithm and are colored according to conservation. **e**. Important regions, SNVs and Indels are marked with boxes

**Fig. 4:**
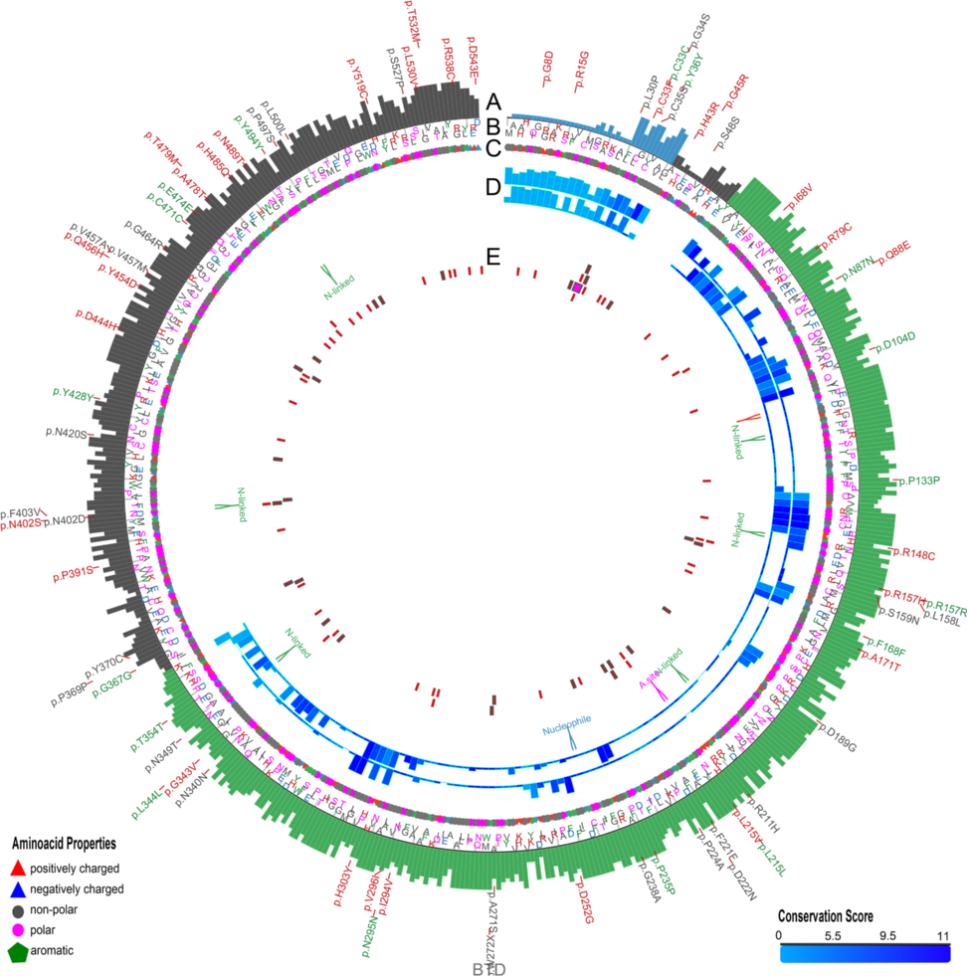
Plot showing conformational differences in *Btd* ^*R*209*C*^ and *Btd* ^*H*447*R*^. Outermost layer indicates reported SNVs (gray, not validated; red, non-synonymous; green, synonymous) from dbSNP. **a**. Conservation scores represented as a histogram (blue, signal peptide; green, CN-hyrolase domain). These values are generated as described in “MODICT methodology” section and are not related to MODICT score. **b** and **c**. Amino acid sequences with residues colored according to their property (positively charged, *red triangle*; negatively charged, *blue triangle*, non-polar, gray circle; polar, pink circle; aromatic ring, green hexagon). **d**. Iterative MODICT scores of individual residue pairs (algorithm, Eq. 1) resulting from comparison with *Btd* ^*WT*^. Top layer belongs to *Btd* ^*R*209*C*^ whereas bottom layer belongs to *Btd* ^*H*447*R*^. Each *blue* histogram bin designates the contribution of a residue pair to the overall MODICT score (Higher bars mean more contribution as well as more the adverse effect of that residue pair on structural stability). These histogram bins are generated by iterative MODICT algorithm and are colored according to conservation. Only scores belonging to domain regions re shown. **e**. Important regions, SNVs and Indels are marked with boxes. (A.site = active site)

**Fig. 5:**
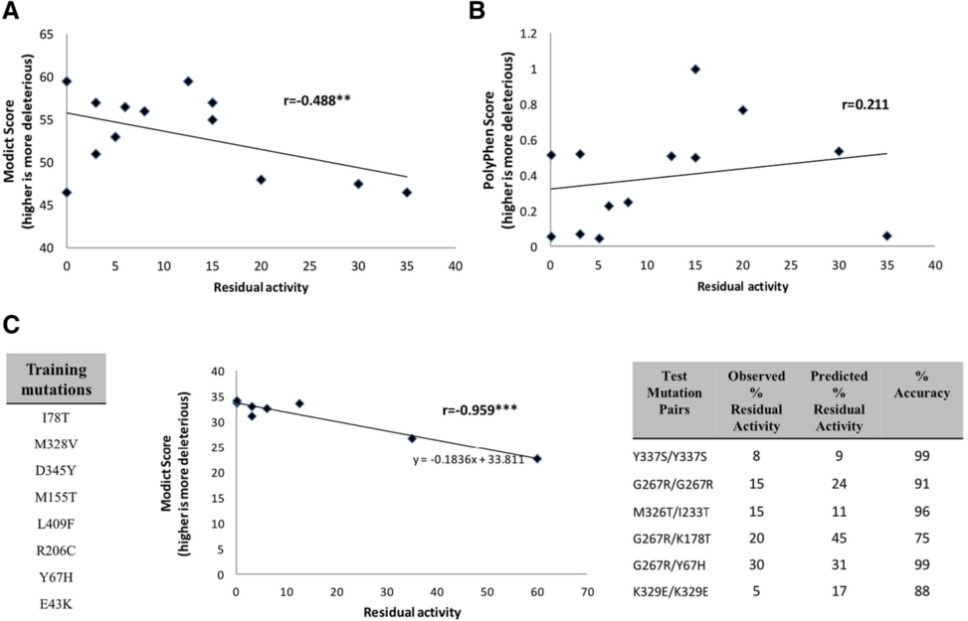
MODICT scores of *ACADM* mutations. **a**. Mutation pairs were plotted based on their enzymatic activity and the average of their MODICT scores. MODICT scores or residual activities that are 2 standard deviations away from the data average was excluded which corresponded to exclusion of only 1 data point (residual activity 60, modict score 53.5). The remaining data points had a correlation coefficient of –0.488 with a *p*-value of 0.044 according to 1 tailed t-distribution. **b**. Same mutations were plotted with POLYPHEN2 scores instead which yielded a positive correlation coefficient of 0.211 with *p*-value of 0.244. **c**. Eight out of 14 mutation pairs in table 2 harbored a p.K329E variant where homozygotes for this mutation only had 5 percent of wildtype activity. Assuming significant portion of residual activity coming from the other variants, these 8 variants (*lower left*) were used as a training dataset for MODICT. After training, MODICT was able to find a weight score combination with a correlation coefficient of –0.959 (*lower mid*). Using the trendline obtained by least squares method, the residual activity of 6 other mutation pairs (that did not include the trained mutations) were guessed. MODICT was able to achieve 91 percent accuracy (*lower right*). (∗∗=*p*<0.05;∗∗∗=*p*<0.001)

**Fig. 6:**
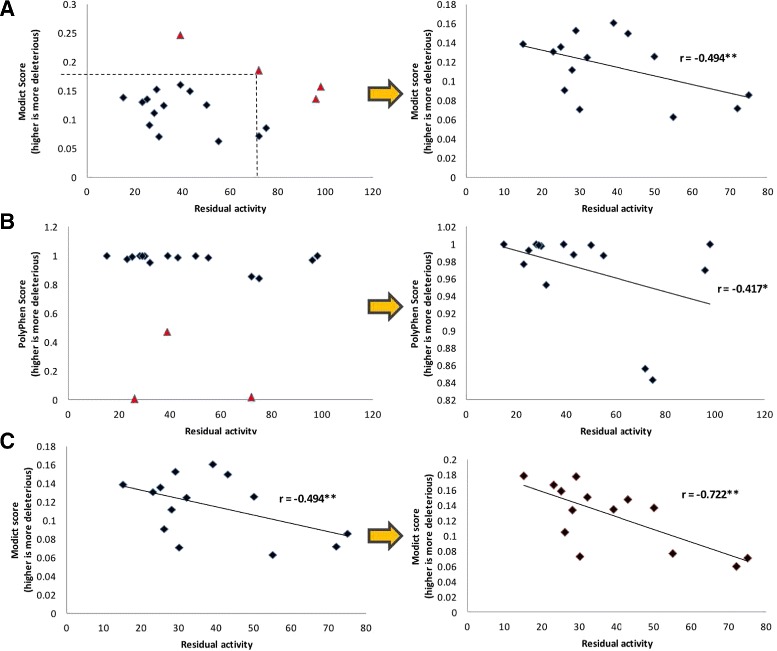
MODICT. SCORES FOR PARTIALLY DELETERIOUS PAH MUTATIONS. *Top Left*. Mutations with residual activity in PAH with their respective MODICT scores are plotted. Triangles indicate data points that are 2 standard deviations apart from the mean (both residual activity and MODICT score) of rectangle data points. *Top Right*. Outliers that are two standard deviations apart from the mean are removed and the correlation coefficient is calculated. MODICT scores are negatively correlated with residual activity (*r*=−0.494). The exact *p*-value of the correlation coefficient is 0.036 based on 1-tailed t-distribution. *Middle Left*. The same comparison was applied to POLYPHEN2 scores. Triangle data points indicate the outliers. *Middle Right*. Likewise, POLYPHEN2 scores were negatively correlated with residual activity (*r*=−0.417). However, the exact *p*-value of the correlation coefficient was 0.062 based on 1-tailed t-distribution. *Lower Left*. The training module of MODICT were used on the same mutations. *Lower Right*. The training module of MODICT was able to achieve a weight score configuration that yielded a more significant *p*-value of 0.002. (∗=*p*<0.1;∗∗=*p*<0.05)

As will be described throughout the “Results” section, MODICT is designed to work with distinct domains which are critical for protein functionality. Often however, this information is not readily available. In order to meet these needs, MODICT comes with a training and iteration module where a random number approach is used to approximate a good candidate weight score combination as in Figs. 2, 3, 4, 5 and 6.

The training module accepts a list of paired MODICT scores and enzymatic activity (or any measure of residual protein function that is determined experimentally). It tries to find an optimal weight score combination for each residue that yields the highest possible Pearson’s correlation (one would expect enzymatic activity and MODICT scores to be negatively correlated). The user has control over the iteration process by regulating several parameters such as the number of rounds to iterate. Even then, improvement of initial correlation varies from protein to protein and depends on the number of mutations to be trained with.

**Fig. 7:**
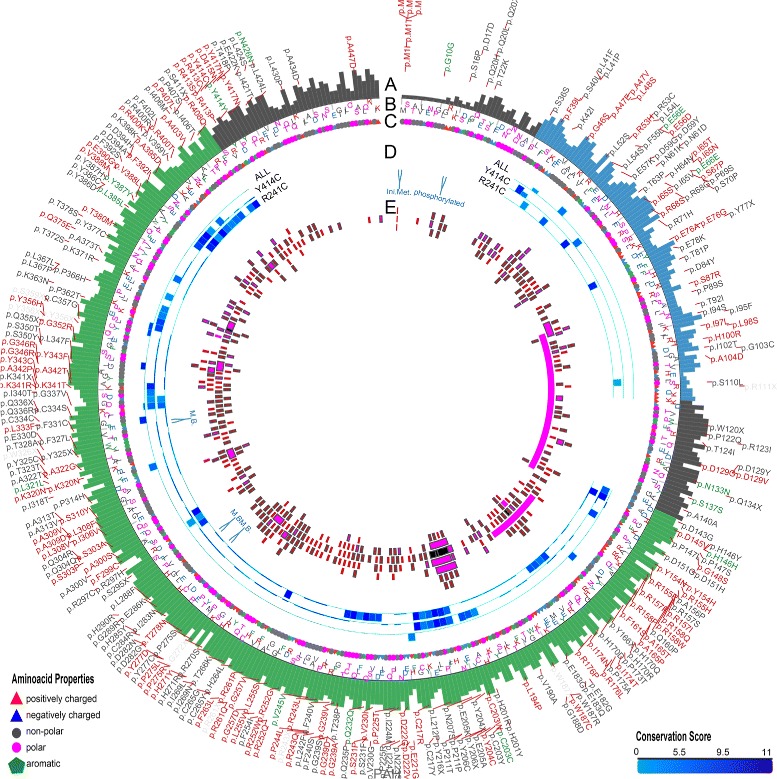
Plot showing conformational differences in *PAH* ^*E*390*G*^, *PAH* ^*V*245*A*^, *PAH* ^*D*415*N*^, *PAH* ^*R*408*Q*^, *PAH* ^*Y*414*C*^ and *PAH* ^*R*241*C*^. Outermost layer indicates reported SNVs (gray, not validated; red, non-synonymous; green, synonymous) from dbSNP. **a**. Conservation scores represented as a histogram (blue, ACT domain; green, catalytic domain). These values are generated as described in section 5 and are not related to MODICT score. **b** and **c**. Amino acid sequences with residues colored according to their property (positively charged, *red triangle*; negatively charged, *blue triangle*, non-polar, gray circle; polar, pink circle; aromatic ring, green hexagon). **d**. Iterative MODICT scores of individual residue pairs (algorithm, Eq. 1) resulting from comparison of mutations with residual enzyme activity less than 50 % (*more severe*) against mutations with residual activity greater than 50 % (*less severe*, Table 1). Each *blue* histogram bin designates the contribution of a residue pair to the overall MODICT score (Higher bars mean more contribution as well as more the adverse effect of that residue pair on structural stability). These histogram bins are generated by iterative MODICT algorithm and are colored according to conservation. Single residue pairs with high blue bars are much less significant than consecutive "blocks" of high blue bars. Scarcity of these blocks in topmost layer (label: all) points to the fact that different regions are affected in each mutation. *PAH* ^*Y*414*C*^ and *PAH* ^*R*241*C*^ are compared to less severe mutations individually (middle and bottom layers). Note the differences in regions that are affected the most in each mutation. **e**. Important regions, SNVs and Indels are marked with boxes

MODICT package also comes with an iterator module to identify regions of a protein that contribute the most to the overall MODICT score (Figs. 2, 3 and 4). The iteration algorithm automatically attains weight scores between 0 and 10 to residues: the higher the weight score, the more the contribution of that residue pair to the overall MODICT score. MODICT uses a random number approach to approximate a significant combination. Although the computation process can be cumbersome under certain conditions, current approach performs well with comparison of many models simultaneously. Such an example is given in Fig. 7 where mutations that preserve more than or equal to 50 percent of residual activity are compared to two relatively more severe mutations.

**Fig. 8:**
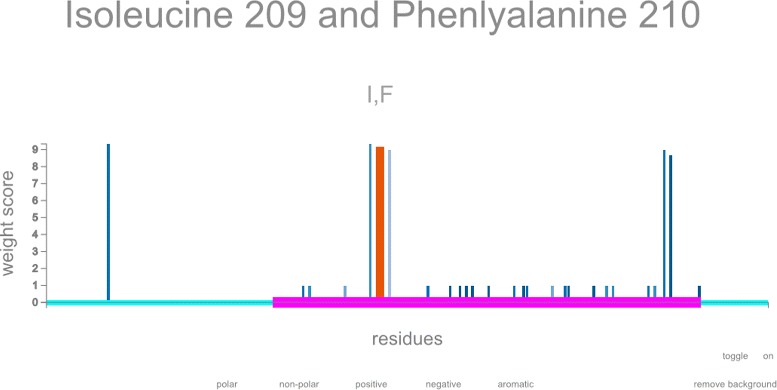
Automatically generated interactable output of iterative MODICT scores. Individual MODICT scores of residue pairs are plotted along the protein with an interactable interface. Annotation data is automatically stored with the use of MODICT. Histograms are automatically colored according to conservation data. Amino acids with different properties can be displayed separately. *Pink* regions highlights the functional domain. Data is taken from comparison of *PAH* ^*Y*414*C*^ against *PAH* ^*E*390*G*^, *PAH* ^*V*245*A*^, *PAH* ^*D*415*N*^ and *PAH* ^*R*408*Q*^. Only the amino acids with aromatic ring is displayed. Mouse over amino acids (209 I and 210 F) are highlighted. For a more comprehensive explanation of how to interpret iterator results please refer to MODICT documentation

When the iteration algorithm of MODICT is used, it generates an automatic and interactable output as shown in Fig. 8. The user can choose to display amino acids with certain properties or just visualize the change in regions that correspond to a domain. The user may wish to know if residues with high MODICT score are also conserved which can be seen from the color coding. For a more comprehensive explanation of how to interpret iterator results please refer to MODICT documentation.

### ROC curve generation

**Fig. 9:**
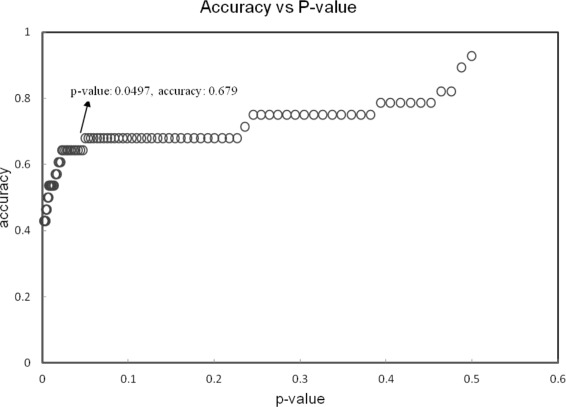
ROC curve. Trio groups (negative control, test, positive control) are tested for decreasing levels of stringency measured as a parameter depending on the standard deviation of the negative controls and the positive controls. There is a trade off between the *p*-value and the stringency. As stringency decreases, accuracy increases, however the increase in accuracy can be explained progressively less by the measurements of the algorithm (increasing *p*-value and decreasing significance). The data used to generate the above plot is indicated in Additional file 1: Table S1. The script for generating the data above is included in MODICT package

One of the challenges to construct a receiver operating characteristic curve (ROC) for an algorithm that generates a continuous range of output rather than a qualitative output (deleterious or benign) is to build a parametric classification system. This can be achieved by recalculating thresholds for a given set of mutations with known outcome while varying the levels of stringency (a measure of how rigorous the thresholds are constructed). Subsequently, this can be plotted against the *p*-value (a measure of how correctly the mutations are classified). In principle, mutations are not only completely benign or deleterious but spread through a range of variable residual protein activity/function. In addition to a negative control which is usually *Δ* RMSD between wildtype and a refined wildtype model or wildtype and a benign model, another score from *Δ* RMSD between wildtype and a given benign/deleterious/partial model should be used. This allows the user to construct a hypothetical distribution of scores and thus determine the likelihood of a test score being benign, deleterious or partial. Such a script is included in the MODICT package. The user can import his calculated scores from new models and update the current ROC plot shown in Fig. 9. Data used to generate the plot is listed in Additional file 1: Table S1.

### Output

MODICT, supplied with the rmsd file, gives as an output an algorithm score, which is a float value without units. A higher score means an increase in probability of a mutation being deleterious. Interpretation of the scores should be based on relative comparison. For instance, when a known pathogenic mutation in a specific protein is run through the MODICT pipeline, a score will be generated which can then be used as a reference. Conversely, this can also be done with a non-pathogenic mutation. This is further exemplified in “Results” section.

## Results

We have derived a simple algorithm MODICT to predict whether a mutation is deleterious or not based on the RMSD obtained from superimposed mutated and wildtype 3D structures. The 3D protein structures in this study were modeled by I-TASSER and PHYRE2, however other modeling algorithms can be used as well. MODICT is not limited by commercially available modeling servers, any set of molecular dynamics simulations can be used. The mathematical model underlying MODICT can also incorporate the information from conservation and weight scores. A default fall back value is provided in case these values are not present. An iteration algorithm to determine the regions that account the most for the calculated score is also available with MODICT. MODICT is not only a prediction tool, but also a tool to scrutinize changes in the protein structure independent of the score.

The algorithm was tested on 6 different proteins which belong to different protein families. The chosen mutations were of different nature in order to minimize bias. Mutations with known phenotypes were chosen which enables to monitor whether if the MODICT scores resonate with real life observations. Most of the mutations come from enzymes in metabolic pathways where enzyme activity can be measured in the patients. This allows one to observe the correlation between MODICT scores and measured enzyme activities. MODICT scores were interpreted by two methods, either correlating them with experimental metrics like enzymatic activities, or using the scores for ordinal classification (deleterious, benign, partially deleterious etc.). The first method requires MODICT scores for at least 3 mutations with experimentally verified enzyme activities for predicting the effect of unknown mutation. Then, the MODICT scores and the enzymatic activity of the known mutations are plotted in a scatter plot and a trend-line is set by the least squares method. By observing the trend-line the enzymatic activity of your mutation of interest can be traced. The advantage of this approach is the ability to use the training module on MODICT for a subset (or the entire set) of mutations to increase the initial Pearson’s r correlation coefficient. This method was applied on Btd, Pah and Acadm mutations (see Tables 1 and 2 and “Btd p.H447R and p.R209C” section).

The second method is used when there are less than or equal to 2 mutations. However a negative control MODICT score is required for comparison. This method was applied on Renin, Tubb2b and Smpd1 mutations (see “Renin p.R33W”, “Tubb2b p.A248V and p.R380L” and “Mutations in Sphingomyelin phosphodiesterase-1” sections). Regardless of the method, higher MODICT scores mean more deleterious.

Throughout this paper MODICT scores have both been used as ordinal classifiers (benign, partially deleterious, deleterious etc.) and continuous variables to measure correlation. In all of the tested cases in this study whether conservation scores and/or weight scores were used or not is indicated. Concerning the examples given in this article, MODICT performs better without conservation scores.

**Fig. 10:**
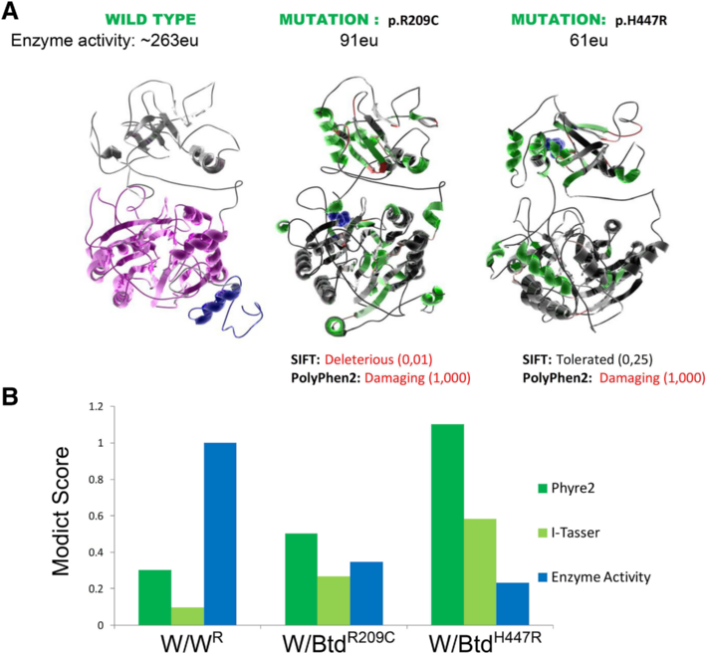
3D models of wildtype and mutated biotinidase. **a**. 3D biotinidase model generated by I-TASSER (A, left). *Pink* residues (57–363) designate the CN-Hydrolase domain whereas the *blue* residues (1-41) designate the signal peptide. Effect of p.R209C and p.H447R mutations on protein structure (**a**, middle, right). *Btd* ^*WT*^ (*left*) is compared to p.R209C (*middle*) and p.H447R (*right*) in means of changes in secondary structure (no change, black; helix to strand, light green; strand to helix, dark green; helix to coil, light red; strand to coil, dark red; coil to strand or helix, green). The mutated R209 and H447 residues are depicted with blue Van Der Waals radii and their POLYPHEN2/SIFT scores and residual enzyme activity are indicated. Comparison of MODICT scores and residual enzyme activity, **b**. MODICT scores from models generated by I-TASSER (negative control, 0.096 ; p.R209C, 0.266 ; p.H447R, 0.584) and PHYRE2 (negative control, 0.301 ; p.R209C, 0.504 ; p.H447R, 1.102) were compared with experimentally measured enzyme activity (wildtype 263eu, p.R209C, 91eu, p.H447R, 61eu) scaled to 1. Ratios of MODICT scores and [1/enzyme activity] are in concordance with each other. (*W* = wildtype, *W* ^*R*^ = refined wildtype)

Throughout the results section, output of the iteration algorithm (residues that contribute the most to a MODICT score) was represented using I-PV as shown in Figs. 2, 3, 4 and 7 [25, 26]. For comparison with other sequence based algorithms, refer to Figs. 5, 6 and 10 and Additional file 1: Table S2. No meaningful correlation could be observed using the SIFT algorithm for the tested mutations.

### Renin p.R33W

Renin is one of the main components that regulates the main arterial blood pressure via the renin-angiotensin system and is initially secreted as a propeptide with a 67 amino acid long signal sequence [27]. Mature renin does not have this signal sequence and is 37kDa long [28]. A novel heterozygous mutation c.58T>C (p.C20R) was found in all affected members of a family with autosomal dominant inheritance of anemia, polyuria, hyperuricemia and chronic kidney disease [29].

**Fig. 11:**
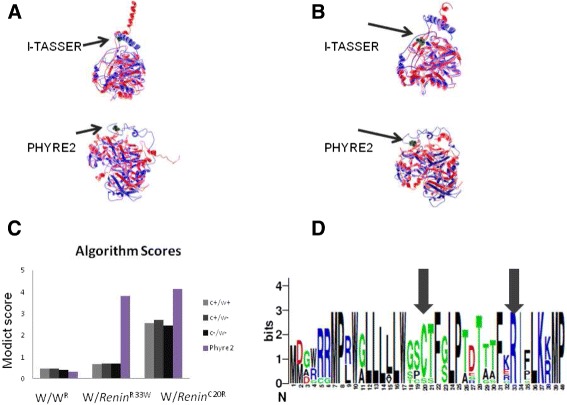
3D models of wildtype and mutated Renin. **a**. Wildtype (*blue*) and *Ren* ^*p*.*C*20*R*^ (*red*) models are superimposed with the cysteine residue (*green*, Van der Waals) marked with arrow. Models generated with different modeling algorithms are indicated. **b**. Another variant in the signal sequence, *Ren* ^*p*.*R*33*W*^ (*red*) does not result in a change to the same extent as *Ren* ^*p*.*C*20*R*^. The wildtype arginine residue (*green*, Van der Waals) is marked with arrow. Graphical representation of algorithm scores, **c**. Absolute values of MODICT scores obtained from pairs; negative control (*left*, light gray; score: 0.455), wildtype against *Ren* ^*p*.*R*33*W*^ (*middle*, light gray; score: 0.670) and positive control (*right*, light gray; score: 2.570). Algorithm scores with or without conservation (c) and weight (w) scores are also indicated (dark gray, black, see Additional file 1: Table S1). For comparison, algorithm scores generated using models from PHYRE2 is also indicated. Like black bars, these are raw MODICT scores generated without conservation and weight parameters. Sequence logo of the renin signal peptide. **d**. Residues 1-40 of reviewed renin sequences in UniProt database have been aligned. Note that both R33 and C20 are highly conserved, however algorithm scores significantly differ in case of I-TASSER. MODICT scores were generated taking into account the main chain (residues 67-406, UNIPROT, P00797). (*W* = wildtype, *W* ^*R*^= refined wildtype)

Another variant p.R33W suspected to be benign resides within the same signal sequence (http://www.ncbi.nlm.nih.gov/projects/SNP/snp_ref.cgi?rs=11571098;- http://web.expasy.org/variant_pages/VAR_020375.html). Several prediction algorithms were tested on this variant previously [30]. In this example, conservation scores generated by multiple sequence alignment of reviewed Ren (renin) sequences were also used by the algorithm as an additional factor (Additional file 1: Section S1.3). Based on domain annotations, residues that are involved in various interactions were also given a weight score of 20 instead of default value (10, Additional file 1: Section S1.3). Figures 2 and 11 c show the algorithm results associated with these mutations.

We also provided wildtype and mutated Renin fasta files to automated PHYRE2 server and received models for the same variants. Wildtype Renin score was 0.328 whereas p.R33W and p.C20R scores were 3.816 and 4.128 respectively. Based on these scores p.R33W variant should be classified as deleterious. As mentioned previously, the p.R33W is of unknown significance due to its low frequency (dbSNP, <1 %). Although a study has claimed that it significantly reduces Renin biosynthesis (http://www.ashg.org/2014meeting/abstracts/fulltext/f140120880.htm), to our knowledge it has not yet been published. The Renin example demonstrates that MODICT scores are not totally independent from the models provided to it. For more detailed explanation for using MODICT scores as an ordinal classifier, please refer to the manual and Additional file 1: Section S1.3.

### Tubb2b p.A248V and p.R380L

Tubulins are the main components of microtubules on which dynein and kinesin motor proteins bind. Together with intermediate filaments and microfilaments, they form the cytoskeleton which plays a major role in intercellular trafficking, cell-cell interactions, junctions and cellular migration [31]. Tubulins are ubiquitously expressed in all human tissues. However mutations in these proteins mostly affect tissue types that rely on their functionality the most during development such as cells of neuronal or glial origin [32, 33]. Almost all mutations in tubulins result in Malformations of Cortical Development (MCD) [34]. Mutations in *TUBB2B* result in polymicrogyria spectrum of malformations [35–41]. Two de novo mutations in Tubb2b, namely p.A248V and p.R380L in 2 unrelated patients of Turkish and Belgian origin and 1 patient of French-Canadian origin respectively were identified and tested for their MODICT scores [36].

**Fig. 12:**
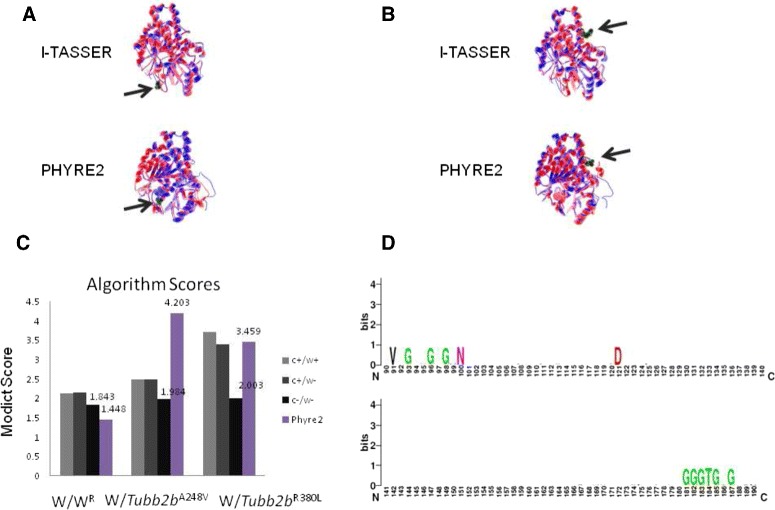
3D models of wildtype and mutated tubulin molecules. **a**. Superimposition of wildtype (*blue*) and *Tubb*2*b* ^*p*.*A*248*V*^ (*red*) models. The alanine residue is rendered with Van der Waals radii (green, gray arrows). Models generated with different modeling algorithms are indicated. **b**. Structural comparison between wildtype (*blue*) and *Tubb*2*b* ^*p*.*R*380*L*^ (*red*) models. The arginine residue rendered with Van der Waals radii (green, gray arrows). Graphical representation of algorithm scores. **c**. Absolute values of algorithm scores obtained from pairs; negative control (*left*, light gray; score: 2.129), wildtype against *Tubb*2*b* ^*p*.*A*248*V*^ (*middle*, light gray; score: 2.485) and wildtype against *Tubb*2*b* ^*p*.*R*380*L*^ (*right*, light gray; score: 3.721). For comparison, algorithm scores generated using models from PHYRE2 is also indicated. Like black bars, these are raw MODICT scores generated without conservation and weight parameters. **d**. Sequence logo of conserved Tubb2b regions. Residues 91-100 and 139-144 of Tubb2b have been conserved since their divergence from the FtsZ proteins. Consequently, during algorithm calculations they have received a weight score of 20 instead of default value. Scores with/without conservation or weight attributes are indicated in C. MODICT scores were generated taking into account the entire backbone (residues 1-445,UNIPROT, Q9BVA1). (*W* = wildtype, *W* ^*R*^ = refined wildtype, c = conservation, w = weight score)

Figures 3 and 12 c show the algorithm results associated with these mutations. Scores without weight and conservation parameters (Additional file 1: Section S1.4) for wildtype, *Tubb*2*b* ^*p*.*A*248*V*^ and *Tubb*2*b* ^*p*.*R*380*L*^ were 1.843, 1.984 and 2.003 respectively. Choosing the wildtype as control (*S*_*C*_) and *Tubb*2*b* ^*p*.*R*380*L*^ as known deleterious mutation (*S*_*K*_), the threshold *T*_1_ was calculated as $\frac {S_{C} + \frac {2 \cdot S_{K}+3.24 \cdot S_{C}}{5.24}}{2}\cdot 3\cdot \kappa /100\cdot \sigma _{(S_{I},S_{K})}$. The value for *T*_1_ was 1.945 which was lower than the *Tubb*2*b* ^*p*.*A*248*V*^ score (*σ*= standard deviation, *κ*=55). This means that the *Tubb*2*b* ^*p*.*A*248*V*^ mutation is indeed deleterious.

Wildtype and mutated fasta files were provided to the automated PHYRE2 server. MODICT scores in the absence of weight and conservation parameters for wildtype, *Tubb*2*b* ^*p*.*A*248*V*^ and *Tubb*2*b* ^*p*.*R*380*L*^ were 1.448, 4.203 and 3.459 respectively. Choosing *Tubb*2*b* ^*p*.*A*248*V*^ as the known deleterious variant, the *T*_1_ threshold is 3.200 which is lower than the *Tubb*2*b* ^*p*.*R*380*L*^ score. As a result, MODICT scores generated by both I-TASSER and PHYRE2 models agree on the nature of the variants.

### Btd p.H447R and p.R209C

Biotinidase is an enzyme that is encoded by the *BTD* gene. Low enzyme activity interferes with the cycling of biotin and if left untreated, it may lead to neurological and cutaneous issues [42]. In this example, a case with experimentally verified results from 2 patients of southeastern Anatolia origin will be used and compared with MODICT scores [43]. The genotype of the patients in the aforementioned study were c.1330G>C (p.D444H)/c.1340A>G (p.H447R)[patient 1, from a consanguineous family] and c.557G>A (p.C186Y)/c.625C>T (p.R209C)[patient 2, from a non-consanguineous family]. Both former mutations (c.1330G>C in patient 1 and c.557G>A in patient 2) were null mutations meaning that the experimentally measured residual enzyme activity belongs to the latter mutations [42, 43]. The residual enzyme activity in the patients were 61*eu* (enzyme units) and 91*eu* respectively (population mean 263*eu*). MODICT scores were generated using 2 different modeling algorithms (I-TASSER, PHYRE2) and results were compared with residual enzyme activity as shown in Fig. 10 [13, 44]. Conservation scores were generated by aligning reviewed biotinidase sequences from UniProt (*Homo sapiens*, *Rattus norvegicus*, *Mus musculus*, *Bos taurus*, *Takifugu rubripes*) by using Clustal Omega ([45]) and the resulting scores (min, 0; max, 11) corresponding to 1-543 residues of Btd were given to MODICT [46]. Supplying or not supplying the conservation scores do not significantly alter the *score*_MODICT_/*enyzmatic*−*activity* ratios as can be seen from Additional file 1: Table S1.

The MODICT scores were generated by taking into account functionally important regions (residues 57–363, 402–403 and 489–490; UNIPROT, P43251). These functionally important regions can generally be found in UNIPROT. As seen in Fig. 10, both PHYRE2 and I-TASSER scores are proportional to corresponding enzymatic activities. Although there are only 2 mutations, taken together with the negative control score, raw MODICT scores without any conservation or weight files correlate strongly with enzymatic activity (PHYRE2: *r*=−0.805; I-TASSER: *r*=−0.838).

### Mutations in Sphingomyelin phosphodiesterase-1

Sphingomyelin phosphodiesterase-1 is an enzyme (Uniprot ID: ASM_HUMAN) located in lysosomes and responsible for conversion of sphingomyelin to ceramide. Deficits in enzyme activity or reduction in the enzyme concentration result in an inborn error of metabolism grouped under the name Niemann-Pick disease (type A and B) [47, 48]. Several polymorphisms exist that are frequent amongst control populations. One example of such variant is the p.V36A located in the signal sequence. Another variant that is often mistaken as deleterious is p.G506R [49, 50]. Using PHYRE2 to model wildtype, Fig. 1 demonstrates the procedure of classifying the p.G506R mutation. Since the known p.V36A variant is benign (with a score of *S*_*K*_), the *S*_*I*_ score is substituted directly by *S*_*K*_. Based on the calculated thresholds, the p.G506R mutation was correctly classified as “partially deleterious or benign”. The procedure to use MODICT as an ordinal classifier using thresholds is further elaborated in the manual and in the Conclusion section.

### Mutations in medium chain Acyl-CoA dehydrogenase

Medium chain acyl-coa dehydrogenase (MCAD, Uniprot ID: P11310, NP_000007.1) is an enzyme encoded by the *ACADM* gene. MCAD deficiency is one of the most common deficits in mitochondrial *β*-oxidation. MCAD is the enzyme responsible for breaking down medium-chain fatty acids. Deleterious mutations that reduce the enzyme activity result in clinical symptoms such as hypoglycemia, hepatic and neuronal dysfunction [51, 52]. Mature MCAD is a homotetramer with four catalytic pockets [53]. The residue E376 is involved in catalytic activity, whereas residue R256 is involved in complex stabilization [53]. Enzymatic activity data of homozygous/compound heterozygous patients carrying 2 deleterious mutations have been adapted from Sturm et al. as shown in Table 2 [51, 52]. Mutated proteins were modeled using PHYRE2 and superimposed on wildtype MCAD which was generated by submitting wildtype fasta file to the PHYRE2 server. For each mutation pair the MODICT score was the average of the MODICT score of individual mutations (direct summation without average only expands the graph on one axis). Rather than using MODICT as a classifier, the main goal was to see if the MODICT scores correlates with the real experimental measurements. MODICT scores correlated negatively with the enzymatic activities as shown in Fig. 5.

Because higher MODICT scores denote more deleterious effect, as the residual activity increases, it’s well expected for MODICT scores to go down which results in negative correlation. As shown in Fig. 5, the initial Pearson’s correlation coefficient was -0.488. Although not very strong, it is important to underscore that MODICT is the first attempt to achieve such degree of correlation between prediction and experimental outcome from user generated 3D protein models. Figure 5 also compares correlation of POLYPHEN2 scores with enzymatic activity which did not yield significant concordance with experimental results.

Figure 5 also depicts the use of the training module of MODICT. Table 2 lists the compound heterozygous mutations used for correlations in Fig. 5. Eight of the mutation pairs in Table 2 share a near-null deleterious p.K329E mutation where homozygotes for this variant has five percent residual activity. Thus, we have trained MODICT with these eight mutations and then used the trendline (calculated by least squares method) to guess the enzymatic activity of other remaining mutation pairs in Table 2. As shown in Fig. 5 (lower right), MODICT was able to achieve 91 % accuracy. The MCAD example demonstrates the possibility of developing an enzyme specific panel without the need of very large datasets for training of MODICT.

### Mutations in *PAH*

The last example is about pheynlketonurea (PKU), an enzymatic defect that manifests itself with the deficiency in phenylalanine hydroxylase (PAH), a phenylalanine to tyrosine converter with the aid of tetrahydrobiopterin (BH4). It is an autosomal recessive disease with both copies of *PAH* carrying deleterious mutations. The ample decrease in PAH activity results in elevated phenylalanine blood concentration. If the elevated phenylalanine concentration is left untreated, it can lead to mental retardation with structural brain changes visible on a MRI. Deleterious mutations in *PAH* affects variably the level of enzymatic activity. Data regarding such mutations can be found in several studies [54, 55] (Table 1). Comparison of the generated MODICT scores after excluding outliers shows that the scores of individual mutations were negatively correlated with residual enzyme activities as shown in Fig. 6 (Pearson’s r =−0.494). Similarly, POLYPHEN2 scores correlated negatively with experimental measurements but to a lesser degree (Pearson’s r =−0.417). Using the training module for the 14 mutations in Fig. 6 further improved the initial correlation coefficient from –0.494 to –0.722.

## Availability and future directions

### Conclusion

MODICT is an algorithm which predicts whether a mutation is deleterious or not. This is based on the RMSD obtained from superimposing mutated and wildtype 3D protein structures. Modeling was done here by using I-TASSER and PHYRE2, although alternatives can be used as well. The mathematical model underlying MODICT can also incorporate the information from conservation and weight scores. An iteration algorithm to determine the regions that account the most for the calculated score is also available with the package.

There are two ways to make use of MODICT scores. The first way is to convert the scores into an ordinal classification system, which requires a negative control. The second way is to correlate experimental results with MODICT scores as shown in the *BTD*, *MCAD* and *PAH* examples. The bottleneck in this approach is to find several known mutations in the protein of interest with available enzymatic activities or an equivalent measurement. However, this method allows an extrapolation between MODICT scores and residual protein activity. By using the MODICT training module, one can further optimize the linear relationship between MODICT scores and residual enzyme activities. Although overall RMSD values and significance is taken into account by the algorithm, MODICT’s accuracy still depends on the models generated by the user. Unlike POLYPHEN2 and SIFT, MODICT scores are not normalized and vary depending on the length of protein, RMSD values between residues, overall RMSD, regions that are taken into account etc. Therefore individual MODICT scores should not be seen as values indicative of deleterious or benign nature, but should always be interpreted in relation to their negative/positive controls or in relation to known enzyme activities.

### Reporting results with Modict

When reporting results using MODICT, users should provide the parameters they used together with the tool. Several of these parameters are key factors in reproducibility of the results. One of these parameters is the modeling algorithm used (PHYRE2, I-TASSER etc.) and the sequence of the protein submitted to the server. The other parameter is the regions that are taken into account (residue numbers, domains etc.) when calculating the MODICT score. The user should also indicate the conservation and the weight scores used, if any. If the training algorithm is used, than the mutations used for training and the output weight score combination should be reported as well. If the user has followed the ordinal classification method, then she/he should also indicate how the negative control score was generated. Lastly, the users should also indicate the superimposition method used for generating the RMSD values. For example, superimposition based on alpha carbon has been used throughout this article.

### Limitations

MODICT is a tool that is not independent on the models generated by the modeling algorithm of choice. The Renin case is a good example for this where models generated by PHYRE2 and I-TASSER gave different MODICT scores. Moreover, consistency in superimposition techniques used between models and the portion of the protein that is actually modeled (full length protein modeling is usually more reliable than partial modeling of distinct domains) significantly affect the outcome. Many modeling servers also include a confidence key together with the results which are useful to judge the quality of starting models. In general, since the wildtype model will be the main model where test and known mutated models are superimposed on, a low quality model will make it harder to discern between scores. Another issue is that many modeling servers have amino acid limits on submitted fasta files which are generally below 2000. This might make the evaluation of large proteins harder. As modeling algorithms advance, several of these issues will be resolved. Another drawback is that all structural deviations from a given wildtype model is perceived towards the deleterious spectrum whereas in reality there are also gain of function mutations. In that case, it is possible to modify the range of weight scores to include negative values as well.

Last, RMSD measurements are not the only indication of protein stability, structural change and function. Certain changes will most likely not be reflected on RMSD level given the accuracy of today’s state of the art modeling algorithms. However we think that the approach of MODICT can inspire researchers to take a novel perspective at least on the remainder of the cases.

### Future directions

It is important to underline that MODICT has no universal training dataset. This means that the algorithm itself (without any weight or conservation parameters) is able to reflect and capture portion of the physio-chemical interactions that determine the outcome of pathogenicity, at least for the proteins demonstrated in this article. In later stages the conservation scores or more importantly the weight scores can be used to train MODICT on a protein basis. For instance certain combinations of weight scores that yield a higher correlation coefficient for a given enzyme panel can be generated. We planning to train MODICT on variety of proteins and upload the trendlines for each modeling algorithm so the end user would only have to upload his/her mutation’s MODICT score without having to train the algorithm manually.

A systematic database of MODICT scores could be very beneficial for additional variant filtering in Next Generation Sequencing analysis as the utilization of protein structures files is not adequately implemented. We are planning to store user-submitted MODICT scores for this purpose. MODICT is a fully automated algorithm that comes with a variety of scripts to analyze the effects of mutations on protein structure. Unlike most other mutation predictors, MODICT uses.pdb files and can simultaneously compare multiple models for differences in topology. All the models used for this article can be downloaded together with the MODICT package from https://github.com/IbrahimTanyalcin/MODICT.

## Availability and requirements

**Project name:** Modict**Homepage:** i-pv.org/modict.html (ready but no material available yet. All the necessary files are available from Github repo.)**Os:** Linux/Os/Windows**Language:** perl v5**Lisence:** GPL

## Additional file

Additional file 1 Supplementary section [56–58]. (PDF 2120 kb)
