# Supplementary material for: Convert your favorite protein modeling program into a mutation predictor: “MODICT”
Source: BMC Bioinformatics. 2016 Oct 19;17:425. doi: 10.1186/s12859-016-1286-0 (PMC5070100; doi:10.1186/s12859-016-1286-0)
Supplement: Additional file 1 — Supplementary section [56–58]. (PDF 2120 kb) [file 12859_2016_1286_MOESM1_ESM.pdf]

Supplementary Section

S1.1 3D protein models and annotation

Amino acid sequences of wildtype and mutant renin, Tubb2b, Btd and Smpd1 proteins (UNIPROT ID: P00797, Q9BVA1, P43251, P17405) were submitted to the automated I-TASSER and PHYRE2 servers. PAH and ACADM (tables 1,2) were submitted to PHYRE2 server with the intensive mode selected (including wildtype fasta files). The obtained 3D models of renin, Tubb2b, Btd and Smpd1 were energy minimized on deepview-swiss-pdbviewer (http://www.expasy.org/spdbv/ [13, 14]) via 2 cycles of steepest descent consisting of 50 steps each and 1 cycle of conjugate gradient consisting of 200 steps with a minimum energy difference(ΔE) of 0.01kJ/mol together with a harmonic constraint of 100 kJ/mol. Models were further refined using MODREFINER (http://zhanglab.ccmb.med.umich.edu/ModRefiner, [51]). For each query a trio pair was constructed by comparing the ratio of the final scores between wildtype/wildtype-refined, wildtype/test and wildtype/mutated where the first and last components serve as negative and positive controls respectively. Images were post-processed with POV-RAY v3.6 (http://www.povray.org). Models can be downloaded together with the MODICT package. The annotation of mutations in this article is in concordance with the Human Genome Variation Society (HGVS,http://www.hgvs.org/).

S1.2 Using MODICT scores

There are two ways to make use of MODICT scores. The first way is to convert the scores into an ordinal classification system, which requires a negative control. A negative control score is made by superimposing WT-refined over WT pair (see section S1.1). For the first way, the negative control score can be generated by resubmitting your wildtype model to a refinement server such as MODREFINER. Or the user can use his own *in-silico* pipeline for model refinement. After refinement, the user should superimpose the refined wildtype model on the wildtype one to generate a negative control score. The important point here is to apply the same refinement procedure to mutated models before superimposing them on the wildtype. However, to justify the use of this system, the user has to have only 2 mutations (one with known effect) with no enzymatic activities to correlate with. The reason is, if there are multiple known mutations, then there will be multiple thresholds. The second approach yields higher resolution and alleviates the problem of multiple thresholds. Supposing you have 3 MODICT scores (negative control: S_C, test: S_T, any score from known mutation: S_K), it is possible that your known mutation might be deleterious, partially deleterious or benign. The first two cases requires you to reverse calculate an hypothetical benign (S_I) such that $\frac{S_C+S_I}{2} + 3 \cdot \sigma_{S_C,S_I} = S_K$ ($\sigma_{x,y}$ = standard deviation of x and y) for a deleterious S_K, $\frac{S_C+S_I}{2} + \frac{3}{2} \cdot \sigma_{S_C,S_I} = S_K$ for a partially deleterious S_K and simply $S_I = S_K$ for a benign S_K. Than the critical value can be expressed as $S_{Crit} = \frac{S_C+S_I}{2} + 3 \cdot \kappa \cdot \sigma_{S_C,S_I}$. If S_T is greater than S_{Crit}, than the query mutation is classified as deleterious. If the difference $(\frac{S_T - \frac{S_C+S_I}{2}}{\sigma_{S_C,S_I}})$ is between 3κ and 1.5κ standard deviations than the mutation is classified as partially deleterious and finally if the difference is less than 1.5κ, than the mutation is classified as benign. The value of κ is determined from the ROC (receiver operating characteristic; refer to section 2.4) plot with the data listed in table S1 and the current value is 0.55.
The second way is to correlate experimental results with MODICT scores as shown in BTD, MCAD and PAH (see sections 3.3, 3.5 and 3.6) examples. The bottleneck in this approach is to find several mutations in the protein of interest with available enzymatic activities or an equivalent measures.

S1.3 Renin p.R33W

Conservation scores were generated by multiple sequence alignment of reviewed Ren (renin) sequences (Uniprot Entry names: RENI1_MOUSE (*Mus musculus*), RENI_CANFA (*Canis familiaris*), RENI_MACMU (*Macaca mulatta*), RENI_SHEEP (*Ovis aries*), RENI2_MOUSE (*Mus musculus*), RENI_HUMAN (*Homo sapiens*), RENI_PANTR (*Pan troglodytes*), RENI_CALJA (*Callithrix jacchus*), RENI_MACFA (*Macaca fascicularis*), RENI_RAT *Rattus norvegicus*). Domain annotation was based on databases of PROSITE (http://prosite.expasy.org/), INTERPRO ([52, 53]) and UniProt.
Using MODICT as an ordinal classifier requires calculating thresholds. Figure 1 scores are given for algorithm results generated taking into account weight and conservation scores. To focus on results generated solely by MODICT, scores generated without weight or conservation scores will be used which are indicated in table S1 and as black bars in figure 1C. To calculate thresholds, a κ value is also necessary which is generated based on the examples in table S1. Current value of κ is 55 (based on the mutations tested in this article). Users can update table S1 with additional data. In principle, more data points (mutations with known effect) will output a more realistic κ value. Taking the negative control score (S_C) as 0.396, the known mutation score (S_K) as 2.491, an imaginary benign score (S_I) is calculated as $\frac{(2 \cdot S_K + 3.24 \cdot S_C)}{5.24}$. Next, the T_1 threshold is calculated as $(\frac{S_I+S_C}{2}) \cdot 3 \cdot \kappa/100 \cdot \sigma_{(S_I,S_C)}$ which in this case is 1.705 (σ = standard deviation). If your test score is larger than this value, then your mutation is classified as deleterious. The value of p.R33W (0.684) is smaller than this value which requires calculation of threshold T_2 given by $(\frac{S_I+S_C}{2}) \cdot 3/2 \cdot \kappa/100 \cdot \sigma_{(S_I,S_C)}$. The value of T_2 for this case is 1.247 which the p.R33W score is below and thus classified as benign. If the score would be larger than T_2 but below T_1, the variant would be considered as partially deleterious. Values of κ below 66 also enable calculation of T_3 threshold which divides partially deleterious mutations into 2 classes: partially deleterious or benign and partially deleterious. For this example T_3 calculation is not necessary.

S1.4 Tubb2b p.A248V and p.R380L

Conservation scores were generated by aligning reviewed Tubb2b (*Homo sapiens, Mus musculus, Rattus norvegicus, Bos taurus, Xenopus laevis*), Tuba1a (*Homo sapiens, Mus musculus, Rattus norvegicus, Sus scrofa, Pan troglodytes, Cricetulus griseus*), Tubb3 (*Homo sapiens, Mus musculus, Rattus norvegicus, Bos taurus, Macaca fascicularis, Arabidopsis thaliana*) and FtsZ (*M. jannaschii, S. aureus, E. coli*) sequences from UniProt. Moreover, weight scores

were attained based on alignment of FtsZ (*M. jannaschii*, *S. aureus*, *E. coli*) sequences with Tubb2b as shown in figure 3 (D).

S1.5 Additional comments

As shown in figures 1 and 3, it is relatively clear to classify Renin^{R33W} compared to Renin^{C20R}, however differences in the tubulin dataset are relatively small and thus calculation of score brackets is necessary. As a general rule of thumb, proteins that are evolutionarily conserved across species are more sensitive to missense mutations and this fact is reflected on the data by exhibition of closer MODICT scores between different mutations. This phenomenon can be observed by elevation of negative control scores like in figure 3 for the Tubb2b protein.

As previoulsy stated, there are two ways to make use of MODICT scores. The first way is to convert the scores into an ordinal classification system, which requires a negative control. The second way is to correlate experimental results with MODICT scores as shown in BTD, MCAD and PAH examples. The bottleneck in this approach is to find several known mutations in the protein of interest with available enzymatic activities or an equivalent measurements. The advantage of this method is to be able to omit the negative control score as the linear trendline (assessed by least squares method) becomes the main means of calculating predicted enzymatic activities. Another advantage is to be able to use the training module for MODICT. Training MODICT on subset of mutations increase the linear relationship between residual enzyme activities and MODICT scores. Consequently the new trendline can be used to remap enzymatic activities of new mutations as shown in MCAD example, figure 8.

MODICT should be seen as a tool rather than an "all in one" program to predict a variant's pathogenicity. It is an attempt to standardize the usage of user generated 3D models for predicting the effect of mutations. MODICT is licensed under GPL and is composed of 7 scripts and 2 modules which ultimately aim to relate extracted RMSD values from mutated proteins with experimental results. Although overall RMSD values and significance is taken into account by the algorithm, MODICT's accuracy still depends on the models generated by the user. Unlike POLYPHEN2 and SIFT, MODICT scores are not normalized and vary depending on the length of protein, RMSD values between residues, overall RMSD, regions that are taken into account etc. Therefore individual MODICT scores should not be seen as values indicative of deleterious or benign nature; MODICT scores are unit-less. Rather than a universal threshold, the relationship between MODICT scores are important in their interpretation. The two methodologies for interpretation (ordinal classification and correlation) have been shown throughout this article. Comparison of MODICT scores are always done within the same protein. Therefore using large number of mutations from different family of proteins for bench-marking is not relevant in case of MODICT as opposed to mainly sequence-based predictors like POLYPHEN2 and SIFT. This does not mean that information in sequence is obsolete, on the contrary, it means that MODICT allows users to approach the prediction process from a different angle.

Table S1 roc curve data.

Wildtype	Given	Test	$Condition^{test}$	$Condition^{given}$	Conservation	Algorithm	Protein	$Mutation^{given}$	$Mutation^{test}$
0.467	0.704	2.696	deleterious	benign	alignment	I-TASSER†	renin	R33W*	C20R
0.467	2.696	0.704	benign	deleterious	alignment	I-TASSER†	renin	C20R	R33W*
0.396	0.684	2.453	deleterious	benign	default	I-TASSER†	renin	R33W*	C20R
0.396	2.453	0.684	benign	deleterious	default	I-TASSER†	renin	C20R	R33W*
2.158	2.491	3.401	deleterious	deleterious	alignment	I-TASSER†	Tubb2b	A248V	R380L
2.158	3.401	2.491	deleterious	deleterious	alignment	I-TASSER†	Tubb2b	R380L	A248V
1.843	1.984	2.003	deleterious	deleterious	default	I-TASSER†	Tubb2b	A248V	R380L
1.843	2.003	1.984	deleterious	deleterious	default	I-TASSER†	Tubb2b	R380L	A248V
0.092	0.267	0.619	partial	partial	default	I-TASSER†	Btd	R209C	H447R
0.092	0.619	0.267	partial	partial	default	I-TASSER†	Btd	H447R	R209C
0.1	0.272	0.599	partial	partial	alignment	I-TASSER†	Btd	R209C	H447R
0.1	0.599	0.272	partial	partial	alignment	I-TASSER†	Btd	H447R	R209C
6.2	160.269	162.143	deleterious	benign	alignment	I-TASSER	tmem	A198V	G212V
6.2	162.143	160.269	benign	deleterious	alignment	I-TASSER	tmem	G212V	A198V
2.919	67.783	68.283	deleterious	benign	default	I-TASSER	tmem	A198V	G212V
2.919	68.283	67.783	benign	deleterious	default	I-TASSER	tmem	G212V	A198V
0.489	2.176	2.775	deleterious	deleterious	alignment	I-TASSER†	ACADM	E43K	K329E
0.489	2.775	2.176	deleterious	deleterious	alignment	I-TASSER†	ACADM	K329E	E43K
0.467	2.147	2.605	deleterious	deleterious	default	I-TASSER†	ACADM	E43K	K329E
0.467	2.605	2.147	deleterious	deleterious	default	I-TASSER†	ACADM	K329E	E43K
0.33	1.127	0.514	partial	partial	alignment	PHYRE2	Btd	H447R	R209C
0.33	0.514	1.127	partial	partial	alignment	PHYRE2	Btd	R209C	H447R
0.325	1.175	0.562	partial	partial	default	PHYRE2	Btd	H447R	R209C
0.325	0.562	1.175	partial	partial	default	PHYRE2	Btd	R209C	H447R
14.127	217.87	307.33	benign	benign	alignment	PHYRE2	Smpd1	V36A	G506R
14.127	307.33	217.87	benign	benign	alignment	PHYRE2	Smpd1	G506R	V36A
8.56	120.85	135.93	benign	benign	default	PHYRE2	Smpd1	V36A	G506R
8.56	135.93	120.85	benign	benign	default	PHYRE2	Smpd1	G506R	V36A

Each line constitutes a trio composed of a negative control (wildtype), a positive control (given) and a test. The results of all mutations are previously known and are written under conditions column. Different modeling algorithms are used to minimize bias and they are indicated. Proteins functional in its entirety are tested along the protein backbone whereas proteins with known domain annotations are tested for specific regions. (*= Clinical significance unknown;no study in favor of adverse functional affect has been published in a scientific journal during the time of this project. †= Also modeled with PHYRE2 as demonstrated in the results section.)

Table S2 Mutations in renin and tubulin.

Algorithm	Mutation	Prediction	Website
ALIGNGVGD	ReninC20R	Less likely to interfere	http://agvgd.iarc.fr/agvgd_input.php
	ReninR33W	Less likely to interfere	
	Tubb2bA248V	Less likely to interfere	
	Tubb2bR380L	Most likely to interfere	
MUPRO	ReninC20R	INCREASED STABILITY	http://www.ics.uci.edu/~baldig/mutation.html
	ReninR33W	INCREASED STABILITY	
	Tubb2bA248V	INCREASED STABILITY	
	Tubb2bR380L	INCREASED STABILITY	
PANTHER	ReninC20R	Pdeleterious: N/A	http://www.pantherdb.org/tools/cnspScoreForm.jsp
	ReninR33W	Pdeleterious: N/A	
	Tubb2bA248V	Pdeleterious: 0,37152	
	Tubb2bR380L	Pdeleterious: 0,83443	
PMUT	ReninC20R	N/A	http://www.mmb2.pcb.ub.es:8080/PMut/
	ReninR33W	N/A	
	Tubb2bA248V	PATHOLOGICAL	
	Tubb2bR380L	PATHOLOGICAL	
POLYPHEN2	ReninC20R	POSSIBLY DAMAGING	http://genetics.bwh.harvard.edu/pph2/
	ReninR33W	POSSIBLY DAMAGING	
	Tubb2bA248V	BENIGN	
	Tubb2bR380L	POSSIBLY DAMAGING	
SIFT	ReninC20R	TOLERATED	http://sift.jcvi.org/
	ReninR33W	Deleterious	
	Tubb2bA248V	Deleterious	
	Tubb2bR380L	Deleterious	
MUTPRED	ReninC20R	Gain of Disorder ($P=0.0401$)	http://mutpred.mutdb.org/
	ReninR33W	Gain of ubiquitination at K37 ($P = 0.0653$)	
	Tubb2bA248V	Loss of helix	
	Tubb2bR380L	Loss of MoRF binding ($p=0{,}0172$)	
SNPS&GO	ReninC20R	DISEASE-RELATED	http://snps-and-go.biocomp.unibo.it/snps-and-go/
	ReninR33W	NEUTRAL	
	Tubb2bA248V	NEUTRAL	
	Tubb2bR380L	DISEASE-RELATED	
MUTATIONTASTER	ReninC20R	Disease-causing	http://doro.charite.de/
	ReninR33W	Disease-causing	
	Tubb2bA248V	Disease-causing	
	Tubb2bR380L	Disease-causing	

Mutations in renin and tubulin were tested with different commercially available prediction algorithms. (N/A = not available)
